# Report on the National Eye Institute’s Audacious Goals Initiative: Creating a Cellular Environment for Neuroregeneration

**DOI:** 10.1523/ENEURO.0035-18.2018

**Published:** 2018-04-18

**Authors:** Marie E. Burns, Beth Stevens

**Affiliations:** 1 Center for Neuroscience and Departments of Ophthalmology and Vision Science and Cell Biology and Human Anatomy, University of California Davis, Davis, CA 95616; 2 F.M. Kirby Neurobiology Center, Boston Children's Hospital and Harvard Medical School, Boston, MA; 3 Stanley Center for Psychiatric Research, Broad Institute of MIT and Harvard, Cambridge, MA 02115

**Keywords:** eye, glia, inflammation, microglia, regeneration, retina

## Abstract

The cellular environment of the CNS is non-permissive for growth and regeneration. In the retina, transplantation of stem cells has been limited by inefficient survival and integration into existing circuits. In November 2016, as part of the National Eye Institute’s Audacious Goals Initiative (AGI), a diverse collection of investigators gathered for a workshop devoted to articulating the gaps in knowledge, barriers to progress, and ideas for new approaches to understanding cellular environments within the retina and how these environments may be manipulated. In doing so, the group identified the areas of (1) retinal and optic nerve glia, (2) microglia and inflammation, and the (3) extracellular matrix (ECM) and retinal vasculature as key to advancing our understanding and manipulation of the retinal microenvironments. We summarize here the findings of the workshop for the broader scientific community.

## Introduction

The era of cell transplantation and neuroregeneration is today becoming more science than science fiction. The formation of laminated retinal organoids in culture (Meyer et al., 2009, 2011; Eiraku et al., 2011; Zhong et al., 2014; Shirai et al., 2016; Wiley et al., 2016; Reichman et al., 2017; Wahlin et al., 2017) and stem cell transplantation studies are increasingly commonplace. However, the next vital step is to integrate these cells efficiently into a neural circuit or microenvironment supporting a circuit that is otherwise losing function. A way to control the cellular environment to promote transplanted cell survival, differentiation, and integration into the surviving retinal circuitry may be most important for the success of regenerative medicine. To tackle the question of how to create a cellular environment for neuroregeneration, workshop participants were charged to consider (1) the role of astrocytes and microglia as positive or negative mediators in regeneration and synapse formation, (2) the importance of vasculature and extracellular matrix (ECM) in regeneration, and (3) the use of scaffolds and other extrinsic tools to support regeneration and reconnection of retinal neurons.

**Figure 1.**
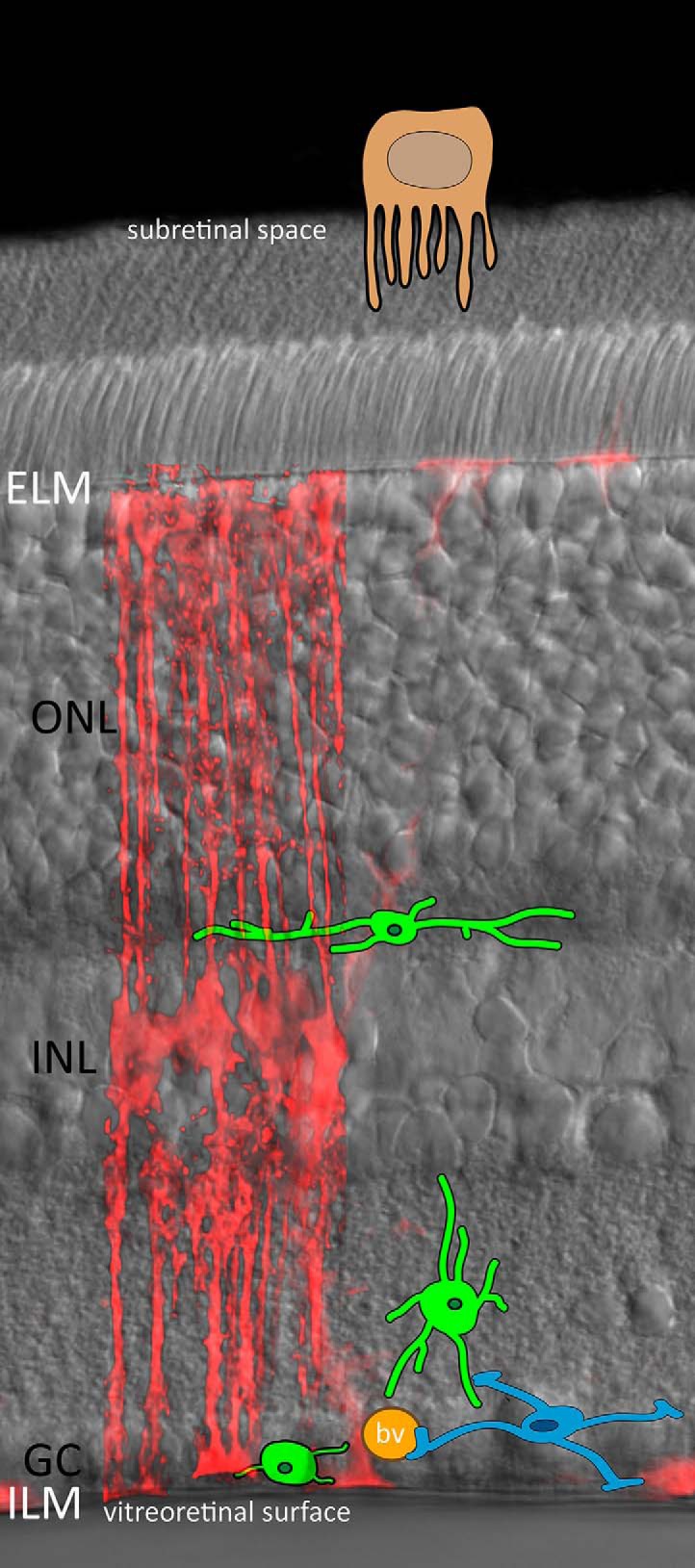
Schematic of local microenvironments within the retina, based on a fixed vibratome section of a mouse retina expressing a viral construct (Shh10-GFAP-mCherry) driving mCherry expression in Müller glial cells (red). Microglia (green) are localized in the synaptic layers between the outer nuclear layer (ONL) and inner nuclear layer (INL) as well as the ganglion cell layer (GC). The subretinal space is subtended by RPE cells (beige) and the ELM secreted by the Müller cells. The Müller cells also secrete the matrix constituting the ILM, which serves as a barrier at the vitreoretinal surface. Astrocytes (blue) are abundant in the nerve fiber layer, where they interact extensively with GCs and blood vessels (bv, orange).

To begin to address these issues, it is first important to recognize some of the unique features of the retina and its place in the posterior eye that present physical barriers and create distinct areas within the neural tissue that define unique environmental subcompartments (Fig. 1), especially with regard to lamination of the retinal tissue. For example, the distal-most, or outermost layer of the retina is comprised of rod and cone photoreceptors, whose survival and disk morphogenesis depends intimately on the overlying retinal pigment epithelial (RPE) cells, the retinal choroid plexus, and beyond these, a very important component of the blood-retinal barrier (BRB), Bruch’s membrane. The proximal retina likewise has a clear anatomical barrier, the internal limiting membrane (ILM), which is composed of collagen and other ECM components secreted by the Müller cell endfeet, which keep the ionic and protein milieu of the CNS distinct from that of vitreous, a boundary known as the vitreo-retinal surface. Further, the optic nerve head, where unmyelinated ganglion cell axons exit the eye in forming the nerve proper, also presents challenges to regeneration. These include age-related changes in the inflammatory microenvironment, especially with regard to microglia, astrocytes, bioenergetic capacity, and oxidative stress.

Within this context, we will first discuss the state of the science regarding retinal glia (Müller cells and astrocytes), the role of resident immune cells (microglia) and neuroinflammation, and the functions of the ECM and BRB. Then we will consider the current best strategies and greatest barriers for progress in these areas. It was clear from the workshop that an improved understanding of how these cells and niches maintain the retinal environment, and how injury and disease perturbs those functions, will enable more successful transplantation and neuroregeneration. Thus, this report adds an additional perspective to those of the previous four Audacious Goals Initiative (AGI) workshops and highlights areas for researchers to address for manipulating the cellular environment so that regenerative strategies have maximal effectiveness.

## Results

### Retinal glia

Glial cells are classically considered the support cells of the nervous system because of their essential role in providing nutrients and growth factors, structural support, maintenance and regulation of the blood-brain and BRB and tissue homeostasis. Glia are particularly adept at sensing and responding to microenvironmental changes. For example, glial expression of aquaporins and TRP channels (Jo et al., 2015) transduce mechanical stress into intracellular calcium signaling and ECM remodeling (Ryskamp et al., 2014; Choi et al., 2015). In recent years, glia have also emerged as active participants capable of refining circuit properties through gliotransmission, promotion of synaptogenesis, synaptic plasticity, and modulation of blood flow through vasoconstriction/dilation in response to changes in electrical activity (Newman, 2015; Giannoni et al., 2018). Elsewhere in the nervous system they also sustain myelination (oligodendrocytes and Schwann cells) and in some instances prevent or impede axon regeneration, either through pro-inflammatory signaling or formation of glial scars (Adams and Gallo, 2018).

The two most abundant glial cell types of the retina, Müller cells and astrocytes, have distinct morphological and physiological characteristics. Müller cells uniquely span all of the retinal layers, from the ILM created by their endfeet at the vitreoretinal surface to the adhesion junctions they make with each other just distal to the outer nuclear layer, called the outer limiting membrane. The distinct, specialized microenvironments of the retina and the subretinal space are unique in their ECM and ionic composition, which the Müller cells help to maintain. Müller cells shuttle potassium ions from the photoreceptors at outer limiting membrane to the vitreoretinal surface, and are essential for maintaining the accompanying changes in osmotic balance (for review, see Sarthy and Ripps, 2001; Bringmann et al., 2006). The intimate contacts between Müller cells and cone photoreceptors also permits retinoid exchange that helps to maintain visual pigment and cone signaling in bright light (Xue et al., 2015). Unlike glia elsewhere in the brain, Müller cells are deficient in pyruvate kinase and rely on metabolites from photoreceptors and other retinal neurons to fuel their mitochondria, one of their many metabolic oddities needed to sustain the demanding outer retinal environment (for review, see Hurley et al., 2015). Müller glia may hold the secret to unlocking neuroregenerative capacity in the retina since they themselves can divide and produce neural progenitors throughout adulthood in fish (Lenkowski and Raymond, 2014) and after injury in chicken (Fischer and Bongini, 2010). This neurogenic state has recently been successfully induced in mice through the ectopic expression in Müller cells of the proneural transcription factor Ascl1 (Jorstad et al., 2017).

Retinal astrocytes are found only in the ganglion cell and nerve fiber layers (Büssow, 1980), where they help to control neural activity, contribute to the integrity of the BRB, and regulate vascular tone (for review, see MacVicar and Newman, 2015). Astrocytes form close contacts with the unmyelinated segments of retinal ganglion cell (RGC) axons, where they may play a particularly important role in supporting the unique metabolic demands of RGCs (Calkins, 2013). Astrocytes show phagocytic activity (for review, see Clarke and Barres, 2013) both in development and in adulthood, with those in the optic nerve head maintaining the extracellular environment by constituitively phagocytosing axons and organelles (Nguyen et al., 2011), including RGC mitochondria (Davis et al., 2014). Because Müller cells are also known for the phagocytic activity (for recent review, see Bejarano-Escobar et al., 2017), both of these classes of retinal (Müller) and optic nerve (astrocyte) glia likely contribute to debris scavenging and microenvironmental homeostasis on an even larger scale than previously appreciated, mimicking to some extent the actions of immune cells that enter the diseased CNS. Failure of these phagocytic roles for glia may promote cell or axon loss over time or inhibit the regenerative capacity of the microenvironment.

In retinal degeneration, like elsewhere in the CNS, glia undergo morphologic changes including stages of hypertrophy and changes in gene expression in a process collectively known as gliosis. Increased expression of the intermediate filament protein, glial filamentous acidic protein (GFAP), is a common hallmark of a late stage of gliosis, but we now appreciate that complex molecular mechanisms likely precede and initiate these profound cellular changes. Gliosis in other parts of the CNS has both adaptive and maladaptive functions. Adaptive functions include regulation and restriction of inflammation and modulation of excitatory activity, whereas maladaptive effects can interfere with the ability of glia to perform their homeostatic functions, making the modulation of different aspects of gliosis a potential therapeutic target (Sofroniew, 2015). More study is needed about such mechanisms in the retina, optic nerve head, and optic nerve proper.

Despite the abundance and central role of glia in retinal health and disease, there are few diseases known to directly result from glial defects. One possible exception is idiopathic juxtafoveolar retinal telangiectasis [also known as macular telangiectasia type 2 (MacTel-2); Gass and Blodi, 1993]. The histopathology of retinas from MacTel-2 patients has demonstrated loss of Müller cells in affected retinal regions (Powner et al., 2010, 2013) ; the mechanistic cause and consequence of these changes remains unknown.

### Microglia and neuroinflammation

In seeking a better understanding of retinal microenvironments and how to manipulate them, there is perhaps no better place to start than endogenous immune cells. Microglia, the resident immune cells of the CNS, constantly surveil their local microenvironment, extending and retracting their branched processes primarily within the synaptic layers of the retina. In response to changes in the immediate milieu (cytokines, complement, extracellular ions like potassium released from damaged cells), microglia transform into migratory, ameboid-shaped cells with greater phagocytic capacity, often referred to as an “activated” state. Activated microglia rapidly respond to sites of injury/degeneration where they engulf stressed or dying cells and debris. In reality, there is an entire spectrum of microglial morphologies between ramified and amoeboid microglial states, making morphologic categorization of limited utility when trying to quantify the degree of neuroinflammation, particularly in chronic diseases with on-going slow degeneration and resolution of inflammation in neighboring regions. Histologically it is sometimes also difficult to discern whether apparent engulfment of neurons by microglia is a manifestation of the high rate of phagocytosis that activated microglia typically display, or whether such contacts might sometimes be providing neurotrophic support. Newly developed approaches for single-cell RNA sequencing, transcriptional and epigenomic profiling of rodent and human microglia, such as DropSeq, is revealing molecular signatures that may help identify specific functional states in health and disease (Butovsky et al., 2014; Matcovitch-Natan et al., 2016; Gosselin et al., 2017; Keren-Shaul et al., 2017).

Microglia, like macrophages, can take on either helpful or harmful roles under different conditions, and the signaling mechanisms that mediate these transitions are poorly understood. In healthy CNS tissue, microglia perform homeostatic and physiologic functions that are crucial for CNS development and for regulating neuroplasticity in the adult. For example, ramified microglia prune unneeded synaptic contacts during development (Paolicelli et al., 2011; Schafer et al., 2012) and mediate experience-dependent plasticity in the visual system (Tremblay et al., 2010). In the healthy adult retina, ablation of microglia leads to a reduction in synaptic transmission and ultrastructural inclusions within photoreceptor synaptic terminals, revealing an essential role for microglia in synaptic maintenance in the retina (Wang et al., 2016). Conversely, inappropriate activation of microglia mediates loss of synapses in a mouse model of Alzheimer’s disease (Hong et al., 2016) and related diseases (Lui et al., 2016; Vasek et al., 2016), reinforcing the view that microglia are homeostatic, microenvironmental biosensors for the CNS.

Interactions between microglia, astroglia, and blood vessels likely help to globally modulate the cellular environment. The Barres Lab (Liddelow et al., 2017) recently showed that when activated, microglia upregulate a number of factors that include TNF-α, IL1α, and C1q; these molecules in turn cause astrocytes to assume a toxic “A1” state in which they produce other factors, as yet unidentified, that lead to the death of RGCs. Simultaneous deletion of the genes encoding these factors or antibodies binding to their protein product lead to strong neuroprotection of RGCs after injury to the optic nerve. Surprisingly, however, another group recently reported that deletion of microglia with the drug PLX5622 does not alter RGC fate after optic nerve injury (Hilla et al., 2017), suggesting that cells other than microglia might also produce the factors responsible for transforming astrocytes. These findings underscore the importance of increasing our understanding of the cellular interactions involving microglia, other inflammatory cells, and astrocytes after CNS damage.

All neurodegeneration appears to involve microglia (Hanisch and Kettenmann, 2007), which can further escalate the immune response by signaling monocytes to extravasate from retinal vessels and migrate to the site of injury. Once in the retinal environment, monocytes rapidly stop expressing some characteristic monocytic markers, like CCR2, and instead express microglial markers (O'Koren et al., 2016), making it difficult to distinguish the fates of these two cell populations during the course of degeneration. These infiltrative neutrophils and macrophages express a host of trophic and cytotoxic factors, of which the small Ca^2+^-binding protein oncomodulin plays a particularly important role in promoting axon outgrowth (Yin et al., 2006). In age-related macular degeneration (AMD) and photoreceptor degeneration, the accumulation of phagocytes in the subretinal space and surrounding drusen can alter the inhibitory, immunosuppressive nature of the microenvironment, leading to disease progression (for review, see Guillonneau et al., 2017). Until recently, it was thought that there was very little turnover of microglia in the healthy CNS over an organism’s lifespan. However, a recent study reported on-going apoptosis and compensatory proliferation (replacement) of microglia in the adult brain (Askew et al., 2017). The differential functions of microglia and monocyte/macrophages in chronic degenerative disease, in age-related susceptibility to disease, and in inflammation following an acute insult is an intense area of research in the CNS in general (Baufeld et al., 2017).

Because microglia play a role in synapse elimination as well as neurodegeneration, they may contribute to synaptic reorganization after transplantation. After optic nerve injury, the survival of RGCs and/or regeneration of their axons is suppressed by the accumulation of mobile zinc in the terminals of amacrine cells and subsequent exocytotic transfer of this zinc to RGCs (Li et al., 2017). During development, RGCs lose their intrinsic ability for robust axonal growth coincident with the time they receive synaptic inputs from interneurons, and cell culture studies suggest that physical contact between RGCs and amacrine cells, the inhibitory interneurons of the retina, play a role (Goldberg et al., 2002a,b). Thus, it may be worth investigating whether synapse elimination or reorganization via microglia or other phagocytic cells normally occurs after optic nerve damage.

### The retinal ECM and the BRB

In the retina, the Müller glial cells are principally responsible for the ECM barriers that construct distinct barriers and microenvironments. Their distal endfeet secrete the collagen-rich basement membrane that constitutes the ILM along the vitreoretinal surface, while their most apical processes envelope the photoreceptor inner segments and form adherens junctions between themselves, forming the external limiting membrane (ELM). Both the ILM and ELM provide structural and diffusional barriers that create microenvironment compartments for the inner and outer retina, respectively (Fig. 1). Within the space between the RPE and ELM, a unique ECM known as the interphotoreceptor matrix (IPM) helps to maintain photoreceptor viability and alignment (Ishikawa et al., 2015). Understanding the distinct composition of these compartments could lead to the control of cell adhesion and motility, affecting both degeneration, repair and regeneration following transplantation. Work in this area is very promising: for example, hybrid scaffolds composed of IPM component and synthetic biopolymers can influence progenitor cell proliferation and promote differentiation toward a photoreceptor-like fate (Baranov et al., 2014).

The molecular details of the matrix comprising the interstitial space in the healthy retina is not well known, though defects in components of the ECM are associated with degeneration and/or functional deficits (for review, see Al-Ubaidi et al., 2013). There may be heterogeneity between the permissiveness of outer and inner retinal matrices, since bipolar cell dendrites can sprout in aging and disease, but RGCs do not. There is also heterogeneity across the surface of the retina, with macular holes healing and filling in, whereas peripheral holes do not. The basis for these differences is not known, but the ECM is likely to be part of the equation.

The retinal blood supply stems from both inner and outer vascular beds: the choriocapillaris, a system of fenestrated capillaries that lies beyond the outer retina and beyond the RPE and Bruch’s membrane, and the retinal vessels emanating from the optic nerve head along the inner retinal surface. The outer BRB is primarily Bruch’s membrane and the tight-junctions between the RPE cells, which prevent the unregulated passage of cells, proteins and ions into the subretinal space. The inner BRB relies on the tight junctions between the vascular endothelial cells themselves, which are affected by inflammation, glycation, and edema (Klaassen et al., 2013). Like elsewhere in the CNS, blood flow and leakage across the BRB is regulated by inner retinal astrocytes, vascular pericytes and perivascular macrophages, and BRB breakdown is associated with many chronic retinal diseases. The molecular mechanisms that maintain a healthy barrier and are affected in disease appear to be somewhat different from those for the blood-brain barrier and remain relatively understudied (Díaz-Coránguez et al., 2017). Two of the most prevalent diseases of the retina, wet AMD and diabetic retinopathy, lead to breaches of the outer and inner BRB, respectively. Thus, the motivation for understanding how the BRB is maintained and how it might be restored in disease is very high.

## Next steps

Many gaps and barriers currently limit our understanding of retinal glia, microglia and inflammation, and the ECM and BRB. First, the role of glia in regulating the retinal microenvironment in both health and disease needs additional study. For example, unlike glia elsewhere in the CNS, there is very little known about the roles of Müller cells at retinal synapses, of astrocytes signaling with RGC axons, and of the normal rate of glial phagocytosis or synaptic maintenance in healthy retina. Are there subtypes or “states” of reactive astrocytes in the retina that exert neurotoxic or neuroprotective effects like in the brain (Liddelow et al., 2017)? Do retinal astrocytes control glymphatic perfusion of the interstitial space like in the brain (Jessen et al., 2015)? To what degree can neuroregeneration be augmented by targeting both astrocyte and microglial activation states?

To tackle these questions as a field, additional tools and resources are needed (Table 1). Although there are several good markers for labeling Müller cells in the retina, including a custom AAV called Shh10 (Klimczak et al., 2009) that drives serotype-specific Müller cell transduction, there is a need for consistent analogous tools for labeling or manipulating astrocytes exclusively in the living retina or optic nerve head. Gene expression profiling of both glial cell types before and during degeneration will help to define the sequence of signaling events that precede frank gliosis, and which may contribute to early loss of neuronal function. Such expression profiling may help to identify drug targets that could in turn be harnessed to control glial function throughout disease progression, both by boosting glial-based metabolic/neurotrophic support and by delaying or reversing gliosis itself. Finally, a turn away from typical rodent-based model systems could dramatically change our understanding of Müller cell function, since Müller cells in lower vertebrates readily regenerate or transform to a progenitor-like state (Goldman, 2014). Emerging research indicates a similar, though far more limited, capacity in rodents, which we may learn to harness by comparisons with lower vertebrates. Understanding this regenerative power is the first step to harnessing its potential for control of the microenvironment and improving transplantation efficiency.

**Table 1.** Specific recommendations from workshop participants

• Development of tools for labeling/manipulating astrocytes in the living retina
• Gene expression profiling of glial cell types during gliosis
• A better understand of Müller cell roles in creating and maintaining ECM and retinal health
• Examination of the transcription factors/signaling mechanisms that control microglial state changes
• Methods to label resident and systemic immune cells acutely in order to examine morphology during disease (ideally suitable for use in humans)
• Further understanding of microglia signaling mechanisms during aging and disease
• Methods to make transplanted cells less adherent to CSPGs at scarred sites
• Further development of ECM scaffolds to promote regeneration or integration of transplanted cells
• The ability to manipulate the blood-retina barrier in a region-specific manner for targeted delivery of therapeutic agents

With regards to microglia, because they are motile and dynamic in both form and function, they offer great potential for cell transplantation and neuroregeneration fields. Since microglia are essential for reinforcing and sustaining proper synaptic connections in healthy tissue, they perhaps could likewise be programmed to prune away the stressed or damaged regions of the retina. Achieving this will require a better understanding of the transcription factors or signaling mechanisms that control microglial state changes. Understanding these signaling networks could also be harnessed to develop therapeutics that could boost microglial support functions and delay phagocytic transformation. Developing such reagents is challenging, in part because of the very nature of microglia’s sensitivity to their environment: microglia steadily transform *ex vivo* into an activated phenotype. To study microglia *in vivo*, high-resolution retinal imaging methods like adaptive-optics scanning laser ophthalmoscopy (AO-SLO) can resolve microglial morphology and branch dynamics across different retinal layers in *Cx3CR1^gfp/+^* mice (Zawadzki et al., 2015). While this genetic approach in mice is experimentally convenient, it would be better to label resident and systemic immune cells acutely, which could then be applied to any species, including human subjects in any state of retinal disease. Unlike nearly all other cell types in the retina, to date there are no known viral serotypes or nanoparticle tagging methods that reliably reveal microglia within their native environment. A better understanding of the signaling mechanisms of microglia, and how they change in aging and disease, might allow us to program resident cells to migrate to and deliver support as needed.

Our understanding of the ECM in the retina is limited. As in other tissues, deposition of chondroitin sulfate proteoglycans (CSPGs) generally create sticky barriers through which cells cannot readily migrate or axons readily penetrate. In injury and disease, gliosis leads to disruption of ECM homeostasis and deposition of CSPGs and other inhibitory glycans (neurocan, aggrecan, versican), which will need to be overcome for transplantation therapies to efficiently lead to cell migration, integration and synaptogenesis (Lau et al., 2013). Currently, best strategies for overcoming CSPGs is to simply digest with matrix metalloproteinase-2 (MMP-2), though this non-specific destruction might be problematic because some matrix scarring can prevent the spread of damage. A better alternative would be to make transplanted cells less adherent to CSPGs, making them “blind” to the scarred site.

Studies which define the properties of the interstitial/ECM environment over the course of development and aging could provide important clues to explain why adult tissue is so anti-regenerative. The retina virtually explodes with proliferation during development, with many more neurons being born than are ultimately needed, creating a wave of apoptosis and synaptogenesis that likely drives up the phagocytic activity of glia and microglia alike. Can we exploit the ECM/microenvironment of the developing optic projection (nerve and retina) to better understand why it is not permissive in adulthood? Can we harness the latent regenerative capacity of Müller cells (Sifuentes et al., 2016; Jorstad et al., 2017) or re-engage a development-like state by tweaking the ECM where it is needed during degeneration? Can we engineer specific ECM scaffolds to promote regeneration or integration of transplanted cells or to allow cell and process motility while preserving the benefits of CSPG barriers to isolate damaged areas? How can a healthy BRB be best supported throughout aging and disease? The ability to manipulate the ECM and the BRB in a region-specific manner could be key to modulate glial reactivity and neuroinflammation in disease, and to deliver therapeutic agents from the bloodstream directly to regions of injury and degeneration.

## Summary

To optimize the cellular environment for neuroregeneration and cell transplantation, much more basic knowledge is needed about individual retinal niches, the composition of the interstitial spaces within the retina (e.g., ECM), the optic nerve head, and the optic nerve and how these spaces are normally maintained by the BRB, by glia, and by microglia. Comparisons of these niches through the course of development and aging will inform us of candidate mechanisms to promote growth-friendly environments for regeneration. Each of the fields of study individually have specific needs and strengths (Table 1), though clearly there is a need for collaboration between experts within different niches to accelerate discovery.

Several overarching principles readily emerged from the group’s consideration of the state of the field *in toto*. First, this general area woefully lacks the same depth of basic mechanistic understanding enjoyed by some other fields. Investigators should consider more basic science research and fewer disease-based studies. The rationale for this is that normal function of glia, microglia and the neurovascular unit is likely to be similar across species, whereas individual diseases may produce uniquely specific changes that render common mechanisms difficult to infer. Second, each retinal niche suffers from a paucity of descriptive or cataloged definitions. For example, each niche needs to address issues of cell-type specificity and heterogeneity, and how these populations change during development, aging and disease, in both animal models and humans. DropSeq and other high-throughput methods that are often beyond the means of a single lab or insufficiently hypothesis-driven for a typical individual investigator award are nonetheless vital for identifying the building blocks on which new hypotheses for mechanism can be generated.

Finally, such building blocks of knowledge would presumably lead to the development of new tools which are vital for monitoring and controlling cellular environments. New and better animal models are needed to bridge our understanding from mice to humans. Centralized, managed database repositories and a nationalized system for donor tissue banks would facilitate resource and data sharing in a manner that would ultimately save time and money and promote collaboration. As one participant pointed out, the field of neuroregeneration needs the “optogenetics equivalent” for tweaking glia and microglia to promote cell survival, tissue remodeling, and synaptogenesis as needed. Through cross-fertilization of fields, collaborative approaches, and investment of resources in basic discovery science, manipulation of the cellular environments to promote regeneration can soon be a reality.

## Synthesis

Reviewing Editor: Kirill Martemyanov, The Scripps Research Institute Decisions are customarily a result of the Reviewing Editor and the peer reviewers coming together and discussing their recommendations until a consensus is reached. When revisions are invited, a fact-based synthesis statement explaining their decision and outlining what is needed to prepare a revision will be listed below. The following reviewer(s) agreed to reveal their identity: David Krizaj, Vadim Arshavsky

Synthesis:

Both reviewers agreed on the timeliness and importance of the topic and thought that the review was well written and insightful. There was a sense that the impact of the report may be further enhanced by emphasizing two topics of relevance: 1. Glial transduction and cellular environment and 2. Issues related to blood-retina barrier. There was an understanding that the manuscript primarily deals with reporting on the discussions that occurred, thus the above is not seen as a major deficiency and marching orders but rather as suggestions which may help balancing the report. Acknowledging these topics to the extent that you choose may be the action required. Specific comments and thoughts voiced by the reviewers are provided below.

Reviewer #1

This is a recap of a recent NEI Audacious Goals Initiative meeting organized around the topic of how knowledge of cellular environments within the retina can be exploited for regenerative medicine. Stem cell transplantation and gene therapy are increasingly important in CNS therapy, but the review argues (convincingly) that for such approaches to be effective, we must first understand the roles of retinal glia, their interactions with vasculature and extracellular matrix, and the role of the innate and systemic immune mechanisms.

The review does a good job in summarizing the recent studies on glial plasticity, inflammatory signaling and linking retinal research to studies in the CNS. Influential reports published in recent years are cited. I particularly liked the last section which focuses on the central role of the matrix in neuronal survival and degeneration, and the Next Steps section which encourages the field to explore the regenerative potential of the retinal tissue. Within this context, the authors might want to include the recent studies on the unique genomic profile of the Muller cell in higher/lower vertebrates and their reprogramming into Muller glia-derived progenitor cells (MGPCs), published by the Reh, Fischer and Raymond labs. The abstract led me to expect a section on retinal stem cell biology, transplantation and regeneration but this was not to be.

For a review that focuses on discussion of retinal cellular environments, one unexpected omission was the absence of discussion of glial transduction of ambient information. We know that retinal neurons and glia use TRP channels, aquaporins and purinergic receptors to sense mechanical stress, regulate water transport, mediate ‘wound healing’ and drive retinal inflammation. The role such transducers might play in the sensing of matrix strain and intraocular pressure is important from the conceptual standpoint (e.g., biomechanics as a key feature of the ‘cellular environment’) and translationally. For example, mechanosensors are required to translate alterations in the ECM and cell-cell interactions into intracellular signaling pathways (such as calcium). It would also be appropriate to discuss, within the broader context of the AGI, the function of visual experience in axonal regeneration, and the key roles of trophic factors and growth-associated genes.

It was difficult to avoid noticing that the article is partial to the work from the Barres lab, with generalized paragraphs on CNS astrocytes and microglia that may or may not, be relevant to the retina. How is Alzheimer's disease relevant to retinal regeneration? As noted above, this seems to have come at the cost of recent research on retinal astrogliosis, Muller-microglial interactions and remodeling.

In summary, this is an interesting, well-written review that presumably reflects the discussion on “retinal cellular environments” filtered by the research interests of workshop participants. While, in the view of this reviewer, the article skirts over key concepts about how retinal neurons and glia sense their microenvironment, it does provides valuable and useful insight into retinal microglia. Amongst other omissions that make the article somewhat incongruent with the title would be the failure to discuss the key roles of biomechanics, cellular ambient sensing, visual experience and dedifferentiation in retinal neurodegeneration and neuroregeneration.

Minor

No pericyte in Fig 1.

Line 92. Because Müller cells are also known for the phagocytic activity (e.g. Egensperger et al., 1996; Francke et al., 2001; Mano and Puro, 1990), see (Bejarano-Escobar et al., 2017) for a recent review,..“ reposition the brackets.

Reviewer #2

This commentary is a report from the NEI workshop that aimed to articulate the gaps in knowledge, barriers to progress, and ideas for new approaches to understanding cellular environments within the retina and how these environments may be manipulated. The report is very clearly written and appears to provide an accurate and detailed summary of major programmatic points and recommendations discussed during this workshop. It will be a very useful guiding document. I am not sure whether any scientific issues are supposed to be brought up upon reviewing this type of a report, but I would like to bring up one point, if appropriate. I found the discussion of the blood-retina barrier being quite rudimentary compared with other topics covered by this report. The only recommendation in regards to the BRB is to develop the ability to manipulate it in a region-specific manner for targeted delivery of therapeutic agents. I think this recommendation is too narrow. Intact BRB plays a critical role in supporting the healthy status of both the retina and the brain. Therefore, an important goal for the future efforts is to better understand the maintenance of healthy BRB and to seek a means to support its healthy status during ageing and development of BRB-affecting pathologies. Currently, the interconnection between the intact barrier and neuronal health is much better understood for the brain than for the retina. For example, a series of papers by Zlokovic and colleagues at USC make a compelling case that breaching the BBB is both a causative factor and a strong predictor of the subsequent development of Alzheimer's disease.
